# Early detection of left fallopian tube carcinosarcoma by transvaginal sonography: a case report and review of diagnostic challenges

**DOI:** 10.3389/fonc.2025.1587411

**Published:** 2025-08-26

**Authors:** Junjie Chang, Wenjun Zhang, Hua Wang, Xin Song, Dan Yu

**Affiliations:** Department of Medical Ultrasound, Taihe Hospital, Hubei University of Medicine, Shiyan, China

**Keywords:** primary fallopian tube carcinosarcoma, malignant mixed Müllerian tumors, transvaginal sonography, case report, imaging diagnosis

## Abstract

Fallopian tube carcinosarcoma (FTCS) is an extremely rare and aggressive malignancy, its nonspecific clinical presentation and anatomical location make preoperative diagnosis challenging, often lead to delayed treatment and poor outcomes. Here, we present a case of early-stage FTCS in a 57-year-old postmenopausal woman who presented with vaginal bleeding. Transvaginal sonography (TVS) played a pivotal role in identifying a characteristic sausage-shaped adnexal mass, which was missed on enhanced computed tomography (ECT). The patient underwent comprehensive surgical staging, including total hysterectomy and bilateral adnexectomy, with pathology confirming FTCS, FIGO stage IA. Immunohistochemical analysis revealed a biphasic tumor with strong P53 positivity and a high Ki-67 index (80%), indicative of its aggressive nature. Despite declining adjuvant therapy beyond two cycles of chemotherapy, the patient remained recurrence-free at 24 months postoperatively. This case highlights the critical role of TVS in the early detection of FTCS and underscores the importance of timely surgical intervention in improving outcomes. We also discuss the diagnostic challenges, pathological features, and therapeutic considerations of FTCS, emphasizing the need for further research to optimize diagnostic and treatment strategies for this rare malignancy.

## Introduction

Fallopian tube carcinosarcoma (FTCS), an aggressive malignancy composed of both carcinomatous and sarcomatous elements, represents a subset of malignant mixed Müllerian tumors (MMMTs) arising from the female genital tract (1, 2). FTCS is exceptionally rare, and its etiology and pathogenesis remain poorly understood. Preoperative diagnosis is particularly challenging, especially in early-stage disease, due to its nonspecific clinical presentation and anatomical location. This case report describes an early-stage left FTCS diagnosed via transvaginal sonography (TVS) and highlights the critical role of imaging in guiding timely surgical intervention.

## Case presentation

A 57-year-old woman (para 4, living 4, menopause at 55) presented with a 10-month history of postmenopausal intermittent vaginal bleeding, from spotting to moderate flow without clots, pain, or discharge. She reported no history of postcoital bleeding or use of hormone replacement therapy. Two years ago, she underwent surgery to remove a malignant lung nodule (sized 2.3 × 1.8 cm, in the left lower lobe) without additional therapy in another hospital. It was confirmed as invasive adenocarcinoma (staged as pT1bN0M0 IA2) with no metastasis. Immunohistochemistry results were TTF-1 (+), P40 (−), and ALK (−). The physical exam revealed a well-healed chest scar, clear lungs, and unremarkable cardiovascular, abdominal, and neurological findings. Gynecological exam findings: Normal external genitalia, patent vagina with slight brown discharge, atrophic cervix with shallow fornices, anteverted and atrophic uterus, and a firm, mobile mass on the left adnexa. An outpatient TVS revealed a mass in the left adnexal area, necessitating further examination to assess the tumor’s nature. She was admitted for further evaluation. Preoperative tests showed no cervical lesions or HPV infection. A cervical biopsy indicated chronic inflammation. Laboratory tests showed mild anemia, while her gynecological tumor marker results are: HE4 at 69.4 pmol/L, AFP at 3.4 ng/mL, CEA at 0.85 µg/L, SCC at 0.72 ng/mL, CA125 at 14.1 U/mL, CA199 at 6.27 U/mL, CA15-3 at 13.4 U/mL, with both Premenopausal and Postmenopausal Rome indices at 14.89.

**Figure 1:**
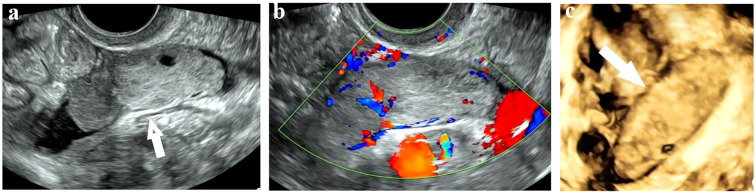
Sonographic images of Fallopian tube carcinosarcoma. **(a)** A solid mass with a clear boundary and regular shape in the left adnexal area. **(b)** Branching blood flow signals in the mass on Color Doppler mode. **(c)** A sausage-shaped mass detected on three-dimensional mode.

Given her lung cancer history, initial tests included enhanced computed tomography (ECT) scans of the chest and abdomen. The chest scan indicated postoperative changes in the left lower lobe and fibrotic foci in both lungs. The abdominal scan showed minor pelvic fluid with no significant abnormalities in the uterus or adnexa. However, Re-examination by three-dimensional TVS revealed a heterogeneous-echoic left adnexal mass (5.9 cm × 2.0 cm × 1.9 cm) with distinct boundaries, an irregular anechoic area, and tortuous, fluid-filled fallopian tubes (**Figure 1a**). Color Doppler imaging demonstrated branching blood flow signals within the mass (**Figure 1b**), and three-dimensional ultrasound revealed a sausage-shaped structure (**Figure 1c**). We consulted the CT diagnostician, who confirmed a sausage-shaped mass with rim enhancement in the left adnexal area upon re-evaluating the images. These findings raised suspicion of a fallopian tube tumor, prompting laparoscopic exploration.

**Figure 2:**
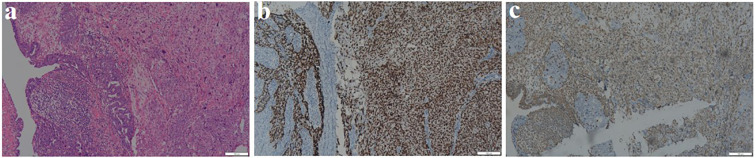
Pathological results of Fallopian tube carcinosarcoma. **(a)** Evident nuclear atypia, pleomorphism, frequent mitoses, and prominent nucleoli seen in the tissue section (HE ×100). **(b)** Immunohistochemical staining shows that both epithelial and **Sarcomatous** components are diffusely and strongly positive for P53 (×100). **(c)** Immunohistochemical staining shows that the Sarcomatous component is positive for Vimentin (×100).

Intraoperatively, the uterus and ovaries appeared normal, but both fallopian tubes were swollen and atretic, with a sausage-like morphology. Intraoperative pathology confirmed a malignant epithelial tumor of the left fallopian tube, with no malignant cells in the ascites. The patient underwent total hysterectomy, bilateral adnexectomy, pelvic lymph node dissection, appendectomy, and omentectomy. Final pathology confirmed left FTCS, FIGO stage IA, with no lymph node metastasis. Right adnexa and bilateral ovaries were free of tumor. Microscopically, the tumor exhibited a biphasic pattern with epithelial and spindle cell components, both showing nuclear atypia, pleomorphism, and high mitotic activity. The malignant spindle cells were closely associated with the epithelial structures, which resembled high-grade serous carcinoma in papillary or glandular patterns, while the sarcomatous elements consisted of dense fascicles of spindle or pleomorphic cells resembling high-grade fibrosarcoma or undifferentiated sarcoma (**Figure 2a**). Immunohistochemistry revealed epithelial differentiation with CKp+, Pax-8+, CK7+, strong P53 (**Figure 2b**), high P16, strong WT-1, weak ER, negative Vimentin and SMA, and a Ki-67 index over 90%. Mesenchymal differentiation showed Vimentin+ (**Figure 2c**), weak CKp, strong P53, negative Pax-8 and ER, weak WT-1, and a Ki-67 index over 80% (**Table 1**).

**Table 1:** Immunohistochemical profile of fallopian tube carcinosarcoma.

Components	CKp	P53	Pax-8	P16	WT-1	Vim	CK 7	SMA	ER	Ki-67
Carcinomatous component	+	Diffuse strong +	+	Strong +	Strong +	–	+	–	Faint +	90%+
Sarcomatous component	Faint +	Diffuse strong +	–	Weak +	Weak +	+	–	–	–	80%+

Postoperatively, the patient received two cycles of paclitaxel-carboplatin chemotherapy but declined further treatment due to no symptoms, normal gynecological tumor markers and unremarkable CT findings. At the 24-month follow-up, she remained recurrence-free.

## Discussion

Fallopian tube carcinosarcoma (FTCS) is an exceedingly rare and aggressive gynecological malignancy, predominantly affecting postmenopausal women with a mean age of 59.7 years, most cases are unilateral without clear side predominance (3). Its clinical presentation is often nonspecific, mimicking primary tubal carcinoma or other gynecologic malignancies, with symptoms such as vaginal bleeding, pelvic pain, or a palpable mass (2, 3). The aggressive nature of FTCS is reflected in its tendency for rapid metastasis and recurrence, as seen in cases where port-site metastasis occurs following laparoscopic surgery (4). The rarity of FTCS and its anatomical location pose significant diagnostic challenges, often leading to delayed diagnosis and poor outcomes (5–7).

The preoperative diagnosis of FTCS is notoriously difficult due to its nonspecific imaging features, and its definitive diagnosis typically requires histopathological examination (6). Despite these challenges, advancements in imaging techniques and surgical interventions have improved the ability to diagnose and manage FTCS. Radiologically, FTCS appears as a “sausage-like” mass along the fallopian tube, unlike ovarian cancer (solid or complex adnexal masses) and endometrial carcinoma (located in the uterine cavity) (8). TVS is advantageous for diagnosing early-stage disease due to its high-resolution imaging of the fallopian tubes and adnexal structures, and its ability to assess blood flow and adjacent organs. It effectively identifies primary fallopian tube cancer features, such as tubular cystic masses with papillary protrusions, cystic solid masses, and “sausage-like” solid masses, often with rich blood flow in protrusions or solid areas (8). In this case, TVS detected a characteristic sausage-shaped mass with hydrops missed by ECT, underscoring the difficulty in distinguishing tubal masses from intestinal tissue and the limitations of CT in early-stage tubal tumors. However, in advanced stages, FTCS often presents as a large, poorly defined mass with metastatic spread, making it difficult to distinguish from ovarian or other pelvic malignancies (5, 7). In such cases, CT or MRI may provide superior diagnostic performance by delineating tumor extent and metastatic spread, with MRI being particularly effective due to its high soft-tissue contrast and multiplanar imaging, and MRI is preferred for diagnosing primary fallopian tube cancer (PFTC) because it can identify key features like a tubular/sausage-shaped mass, hydrosalpinx, and rim enhancement (1, 4, 9). Unfortunately, MRI was not performed in this case, limiting our ability to compare its diagnostic utility with TVS.

FTCS is characterized by a biphasic histologic pattern, comprising both epithelial and mesenchymal components, each exhibiting distinct immunohistochemical (IHC) profile (6, 10). IHC analysis is vital, with FTCS often expressing WT1/PAX8 (indicating Müllerian origin) and vimentin (suggesting sarcomatous differentiation). Care is needed to differentiate FTCS from high-grade serous carcinoma, which also shows WT1 positivity. In this case, the epithelial component expressed CK7, CK-P, and ER, while the spindle cell component was positive for vimentin and negative for ER and S-100. The strong P53 positivity and high Ki-67 index (over 80%) observed in both components are consistent with the aggressive nature of this malignancy and its potential for rapid progression. FTCS can sometimes occur alongside other tumors, for instance, it may coexist with ovary serous carcinoma (11), making it challenging to determine if they are distinct or if one has transformed. In this instance, the patient was diagnosed with stage I primary lung adenocarcinoma and primary FTCS, with no signs of metastasis or recurrence after surgery. This case underscores the importance of early detection and the role of advanced imaging techniques in improving diagnostic accuracy and patient outcomes.

Given the rarity of FTCS, there is a lack of standardized treatment protocols, and much of the current knowledge is derived from case reports and small studies. The primary treatment for FTCS remains surgical resection, typically followed by adjuvant chemotherapy or radiotherapy (7, 10, 12). Genetic analyses have provided insights into the molecular characteristics of FTCS, revealing shared mutations between the carcinomatous and sarcomatous components, suggesting a monoclonal origin and potential pathways for targeted therapy (2). The expression of specific receptors, such as the glucocorticoid receptor, has been studied in carcinosarcomas, indicating potential therapeutic targets that could be explored in future treatments (13). In this case, the patient underwent comprehensive surgical staging, including hysterectomy, bilateral adnexectomy, and lymph node dissection, followed by two cycles of paclitaxel-carboplatin chemotherapy. Despite declining further adjuvant therapy, the patient remained recurrence-free at 24 months postoperatively, likely due to the early-stage diagnosis and complete surgical resection. This favorable outcome highlights the importance of early detection and aggressive surgical management in improving survival outcomes for FTCS patients.

This case highlights the diagnostic and therapeutic challenges associated with FTCS and underscores the critical role of advanced imaging techniques, particularly TVS, in early detection of the disease. While FTCS remains a rare and poorly understood malignancy, advancements in imaging technology and molecular diagnostics hold promise for improving early detection and treatment outcomes. A thorough evaluation using clinical history, radiological features, and extensive IHC profiling is recommended for accurate differential diagnoses. Continued research and collaboration are essential to further our understanding of this disease and optimize patient care.

## Data Availability

The original contributions presented in the study are included in the article/supplementary material. Further inquiries can be directed to the corresponding author.
