# Supplementary figures and images for: Early detection of left fallopian tube carcinosarcoma by transvaginal sonography: a case report and review of diagnostic challenges
Source: Front Oncol. 2025 Aug 26;15:1587411. doi: 10.3389/fonc.2025.1587411 (PMC12417160; doi:10.3389/fonc.2025.1587411)

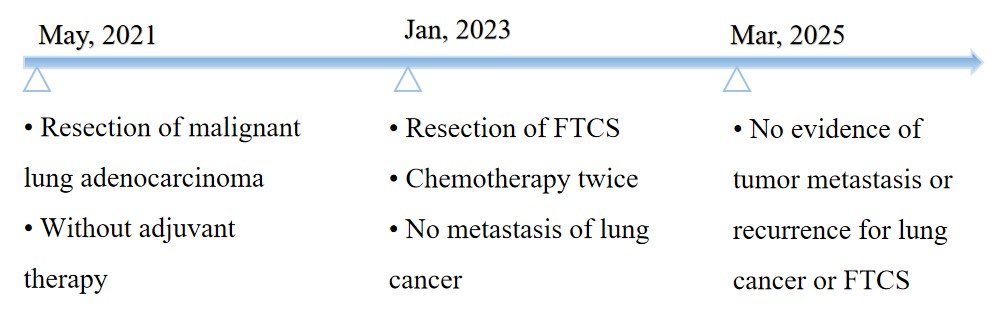

Supplement: Supplementary file 1 [file Image1.jpeg]

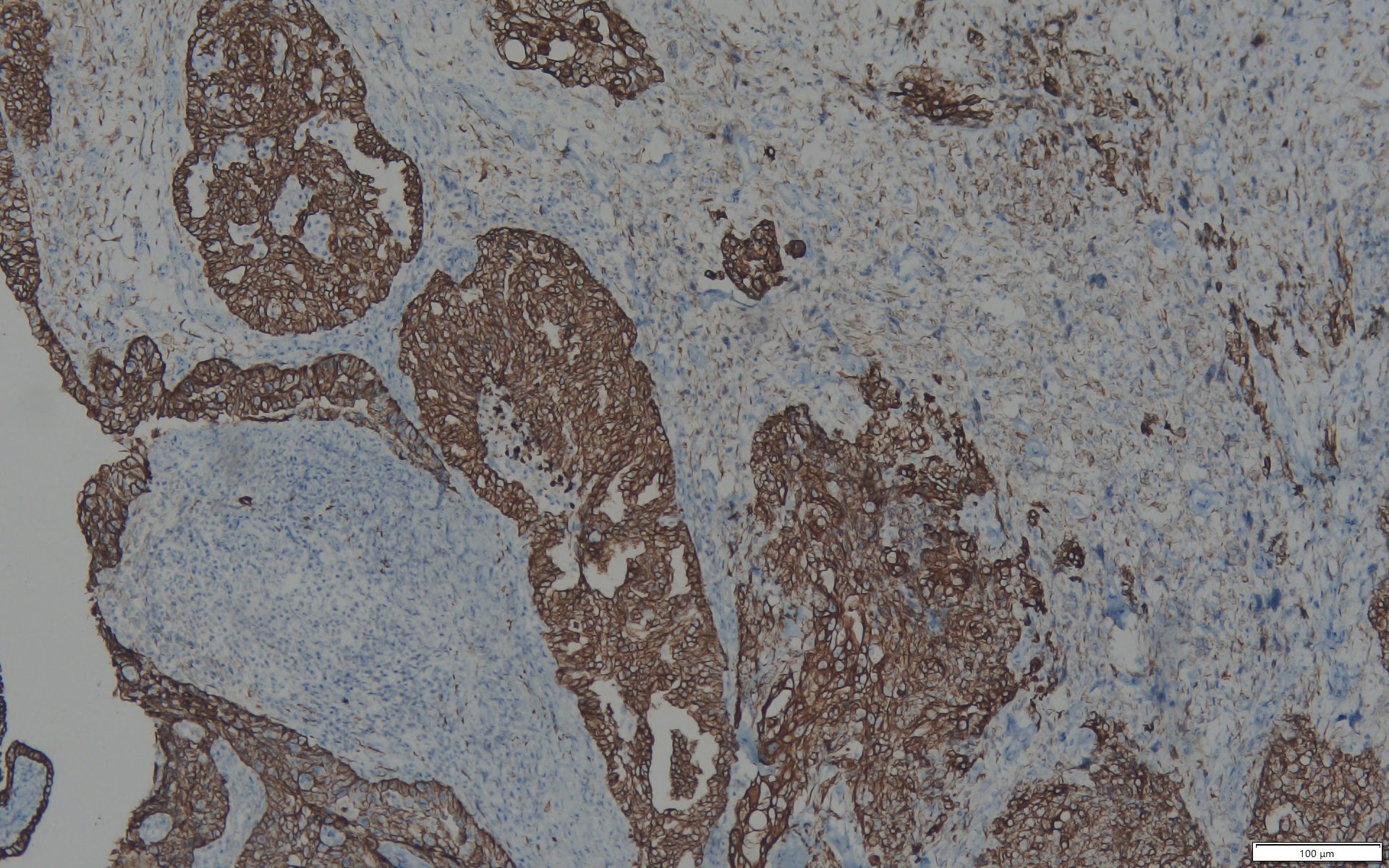

Supplement: Supplementary file 2 [file Image2.jpeg]

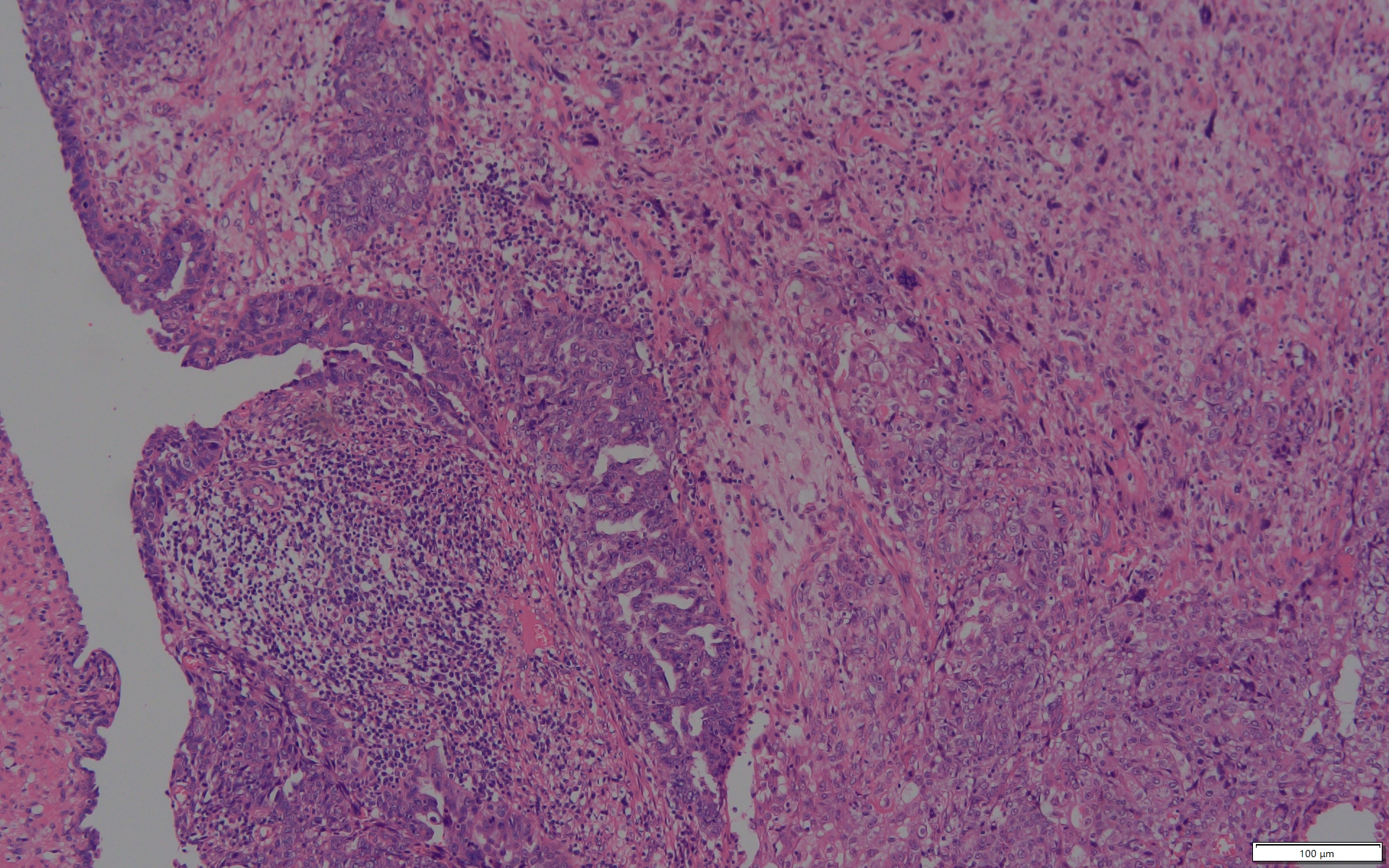

Supplement: Supplementary file 3 [file Image3.jpeg]
